# Iron-induced cytotoxicity mediated by endolysosomal TRPML1 channels is reverted by TFEB

**DOI:** 10.1038/s41419-022-05504-2

**Published:** 2022-12-16

**Authors:** Belén Fernández, Pablo Olmedo, Fernando Gil, Elena Fdez, Yahaira Naaldijk, Pilar Rivero-Ríos, Franz Bracher, Christian Grimm, Grant C. Churchill, Sabine Hilfiker

**Affiliations:** 1 Institute of Parasitology and Biomedicine “López-Neyra”, Consejo Superior de Investigaciones Científicas (CSIC), 18016 Granada, Spain; 2 Department of Legal Medicine and Toxicology, School of Medicine, University of 18016 Granada, Granada, Spain; 3 Department of Anesthesiology and Department of Physiology, Pharmacology and Neuroscience, Rutgers New Jersey Medical School, Newark, NJ 07103 USA; 4 Life Sciences Institute and Department of Cell and Developmental Biology, University of Michigan, Ann Arbor, MI 48109 USA; 5 Department of Pharmacy – Center for Drug Research, Ludwig-Maximilians-University, 81377 Munich, Germany; 6 Walther Straub Institute of Pharmacology and Toxicology Faculty of Medicine, Ludwig-Maximilians-Universitaet, 80336 Munich, Germany; 7 Department of Pharmacology, University of Oxford, Oxford, OX1 3QT UK

**Keywords:** Mechanisms of disease, Cell biology

## Abstract

Increased brain iron content has been consistently reported in sporadic Parkinson’s disease (PD) patients, and an increase in cytosolic free iron is known to cause oxidative stress and cell death. However, whether iron also accumulates in susceptible brain areas in humans or in mouse models of familial PD remains unknown. In addition, whilst the lysosome functions as a critical intracellular iron storage organelle, little is known about the mechanisms underlying lysosomal iron release and how this process is influenced by lysosome biogenesis and/or lysosomal exocytosis. Here, we report an increase in brain iron content also in PD patients due to the common G2019S-LRRK2 mutation as compared to healthy age-matched controls, whilst differences in iron content are not observed in G2019S-LRRK2 knockin as compared to control mice. Chemically triggering iron overload in cultured cells causes cytotoxicity via the endolysosomal release of iron which is mediated by TRPML1. TFEB expression reverts the iron overload-associated cytotoxicity by causing lysosomal exocytosis, which is dependent on a TRPML1-mediated increase in cytosolic calcium levels. Therefore, approaches aimed at increasing TFEB levels, or pharmacological TRPML1 activation in conjunction with iron chelation may prove beneficial against cell death associated with iron overload conditions such as those associated with PD.

## Introduction

Iron is essential for many cell biological processes. It naturally exists either in an oxidized form as ferric iron (Fe^3+^) or in a reduced form as ferrous iron (Fe^2+^). The reduced form of iron is scarce, even though it is the major form of iron used by mammalian cells. Therefore, cells have developed mechanisms for efficient uptake of ferric iron. In serum, Fe^3+^ is bound to the iron transport protein transferrin (Tf) which allows it to bind to the transferrin receptor, followed by receptor-mediated endocytosis [1]. Such receptor-mediated Fe^3+^ uptake also seems to be crucial for neuronal iron acquisition [2]. Once in the acidic lumen of the endolysosome, Fe^3+^ is released from Tf and reduced to Fe^2+^, which then can be transported through endolysosomal Fe^2+^-conducting channels into the cytosol [3]. In the cytosol, Fe^2+^ joins the labile iron pool and is either used in metabolism or stored in ferritin [1]. However, free Fe^2+^ can induce oxidative stress leading to cell death, as it converts hydrogen peroxide to highly reactive hydroxyl radicals via the Fenton reaction. Due to this oxidative effect, the reported accumulation of brain iron in neurodegenerative diseases such as Parkinson’s disease (PD) may contribute to cell death [4, 5].

Endolysosomal cation channels include the TPCN (two-pore channel) and the TRPML (transient receptor potential channel, mucolipin subfamily) channels. Both TPCN and TRPML channels function as Ca^2+^ release channels with roles in endolysosomal transport and fusion processes [6–8]. However, they can also conduct ions other than Ca^2+^ [9–12], indicating that they may play an important role in controlling cation homeostasis in endolysosomal organelles. Altered brain iron handling is observed in humans and animal models of mucolipidosis type IV, a lysosomal storage disease caused by mutations in TRPML1 [13, 14], and TRPML1 has been reported to function as an endolysosomal Fe^2+^-conducting channel [11]. Therefore, under conditions of iron overload as observed in PD, TRPML1 channels may cause cytotoxicity via the release of endolysosomal Fe^2+^ into the cytosol. However, positive roles for TRPML1 in supporting cellular homeostasis have been described as well. Transcription factor EB (TFEB) regulates lysosomal biogenesis and lysosomal exocytosis by inducing the release of endolysosomal Ca^2+^ through TRPML1 [15–18], and TFEB overexpression can prevent cell death in various animal models of neurodegeneration [19–23]. Thus, the effects of TRPML1 channel activity on iron overload-associated cytotoxicity in either the absence or presence of TFEB remain unclear.

Here, we find region-specific increases in brain iron in PD patients due to the common G2019S-LRRK2 mutation, suggesting that brain iron accumulation is commonly observed in both familial and sporadic PD cases. In contrast to human, differences in brain iron content are not observed in a mouse model of the G2019S-LRRK2 mutation. When chemically inducing iron overload in cultured cells, TRPML1 mediates cell death by increasing free cytosolic Fe^2+^ levels. TFEB expression reverts the iron overload-induced cell death by causing lysosomal exocytosis, which depends on increased intracellular calcium levels mediated by TRPML1. Therefore, cell death associated with iron overload may be ameliorated by strategies involving TFEB expression, whilst TRPML1 activation may prove detrimental due to increasing cytosolic Fe^2+^ levels.

## Materials and methods

### Reagents

Reagents included ammonium iron(III) citrate (FAC) (Sigma Aldrich, F5879), 2,2-bipyridyl (Sigma Aldrich, 366-18-7), 4,4-bipyridyl (Sigma Aldrich, 533-26-4) and BAPTA-AM (Invitrogen, B6769). Trans-Ned19 (Ned19) and a cell-permeant version of NAADP (NAADP-AM) were synthesized as previously described [24, 25] and used at a final concentration of 1 μM (Ned19) or 100 nM (NAADP-AM) for 12 h. The TRPML compounds ML-SA1, MK6-83, EVP21, and EVP22 have been previously described [26–29] and were employed at a final concentration of 5 μM for 12 h.

### Human tissue samples

Samples and data from patients included in this study were provided by the Biobank HUB-ICO-IDIBELL (PT17/0015/0024), integrated into the Spanish Biobank Network, and were processed following standard operating procedures with the appropriate approval of the Ethics and Scientific Committees. For each patient and brain area, three different cuts from each frozen tissue block were processed and metal content from each sample analyzed in triplicate by ICP-MS by an experimenter blind to conditions as described below.

### Wild-type and G2019S-LRRK2 knockin mice

Heterozygous breeding pairs of mice [30] were crossed to yield wild-type and homozygous G2019S-LRRK2 progeny as previously described [31]. Homozygous G2019S-LRRK2 were backcrossed to wild-type C57BL6 mice every fourth generation to prevent genetic drift, and genotypes were confirmed by PCR and sequencing. In humans, the G2019S mutation is autosomal-dominant, producing similar disease onset and progression in heterozygous and homozygous mutation carriers [32, 33]. In addition, previous studies showed similar behavioral and elctrophysiological outcomes in heterozygous and homozygous G2019S mice [34–36]. Therefore, we used homozygous G2019S-LRRK2 mice for these experiments. Mice were aged 3–24 postnatal months, and age- and strain-matched wild-type mice served as controls. For each age group, both male and female mice were analyzed. All mice were bred and raised under identical conditions in the vivarium, and all procedures were approved by the CSIC Institutional Animal Care and Use Committee. Mice at the indicated ages were sacrificed, entire tissue samples (cortex and cerebellum) dissected and metal content from each sample analyzed in triplicate by ICP-MS by an experimenter blind to genotype as described below.

### Inductively coupled plasma mass spectroscopy

Tissue samples were weighed and microwave-digested in quartz vessels with 0.5 ml of HNO_3_ (Suprapur, Merck, Darmstadt, Germany) and 1.5 ml of Milli-Q water. The microwave digestion system (Ethos UP, Milestone, Shelton, CT, USA) was programmed at 1800 W and 210 °C as power and temperature limits, respectively (ramp time 20 min; hold time 15 min; cooling time 60 min). The digested solution was then transferred to a decontaminated tube for subsequent analysis. All quartz vessels were vigorously cleaned, soaked for 24 h in 10% HNO_3_, thoroughly rinsed with Milli-Q water, and dried at 80 °C for ~2 h before use.

Quantification of the total Cu, Fe, Mn, and Zn concentrations in tissue samples was performed by ICP-MS on an Agilent 8900 triple quadrupole ICP-MS (Agilent Technologies, Santa Clara, CA, USA) at the laboratory of the Department of Legal Medicine, Toxicology and Physical Anthropology, University of Granada (Granada, Spain). A calibration curve for each element was prepared in Milli-Q water with 2% HNO_3_ (Suprapur, Merck) and 1% HCl (Suprapur, Merck) using appropriate metal standard solutions (Agilent Technologies). All digested samples were diluted 1:20 in Milli-Q water with 1% HCl (Suprapur, Merck) for analysis.

The instrument was tuned and performance parameters were checked prior to analysis. A 400 µg/l internal standard solution with Sc and Ge was added online to the samples to compensate for instrumental instabilities and possible matrix effects. Appropriate blanks were analyzed to correct the results. To ensure the quality of the results, a suitable certified reference material [National Institute of Standards and Technology NIST (Gaithersburg, MD, USA) Trace Elements in Natural Water Standard Reference Material SRM 1640a] was used, and a blank and an intermediate calibration standard were reanalyzed every 12 samples. Additionally, one in every 12 samples was reanalyzed at the end of each session. Tissue metal concentrations were expressed as µg/g wet weight.

### DNA constructs

TRPML1-flag, TRPML2-RFP, and TRPML3-GFP were generous gift from Dr. R. Puertollano (NIH, Bethesda, USA). TRPML1-T232P-flag [11] was generated by site-directed mutagenesis (QuikChange, Stratagene), and the identity of the construct was verified by DNA sequencing of the entire coding region. Human GFP-TFEB was obtained from Addgene (Addgene, 38119) [37].

### Cell culture, transfections, and immunocytochemistry

HEK293T (ATCC, #CRL-3216) and HeLa (ATCC, #CRM-CCL-2) cells were cultured as previously described and regularly tested for mycoplasma contamination [31, 38]. Briefly, HeLa cells were cultured in full medium (DMEM containing 10% fetal bovine serum, non-essential amino acids, and high glucose) at 37 °C in 5% CO_2_. Confluent cells were harvested using 0.05% trypsin and 0.02 mM EDTA in PBS, and subcultured at a ratio of 1:4–1:6. HEK293T cells were cultured in full medium (DMEM containing 10% fetal bovine serum, non-essential amino acids, and low glucose) and subcultured at a ratio of 1:4. In all cases, cells were transfected at 70–80% confluency with Lipofectamine 2000 (Invitrogen, 12566014) according to manufacturer’s specifications for 4 h in DMEM medium, followed by replacement with fresh full medium. Single transfections were performed using 3.5 μg of plasmid of interest and 10 μl of Lipofectamine 2000. For double-transfections, 2.5 μg of each plasmid was employed. Transfected cells were replated the next day at a 1:3 ratio onto poly-l-lysine-coated coverslips (Sigma Aldrich, P2636) in six-well plates, followed by incubation with distinct compounds as indicated before analysis. As appropriate, and to assure that the detected iron was intracellular and not accessible to an extracellular iron chelator, FAC-treated cultures were washed twice with phosphate-buffered saline (PBS, Gibco, 10010023) in the presence of deferoxamine (1 mM; Sigma Aldrich, D9533) [39].

For immunocytochemistry, transfected cells were fixed using 4% paraformaldehyde (PFA) in PBS for 10 min at room temperature, and permeabilized with 0.2% Triton-X100/PBS for 20 min. Coverslips were blocked with 0.5% (w/v) BSA in 0.2% Triton-X100/PBS (blocking solution) for 1 h, followed by incubation with anti-flag antibody (mouse monoclonal anti-flag, Sigma, clone M2, 1:500) in blocking solution overnight at 4 °C. After three washes, coverslips were incubated with secondary antibodies (Alexa488-conjugated goat anti-mouse or Alexa594-conjugated goat anti-mouse; Invitrogen, 1:1000) in blocking solution for 1 h. Unless otherwise specified, images were acquired on a Leica TCS-SP5 confocal microscope using a 63 × 1.4 NA oil UV objective (HCX PLAPO CS). Images were collected using single excitation for each wavelength separately (488 nm Argon Laser line and a 500 to 545 nm emission band pass; HeNe Laser line and a 556 to 673 emission band pass; 405 nm UV diode and a 422 to 466 nm emission band pass). The same laser intensity was used for image acquisition of individual experiments. Images were acquired with a step size of 0.3 μm, and z-stack images analyzed and processed using Leica Applied Systems (LAS AF6000) image acquisition software.

For determination of apoptosis, fixed cells were mounted using mounting medium containing DAPI (Vector Laboratories, H1200) and visualized on a Zeiss microscope (Zeiss International, Goettingen, Germany) using a 100× oil-immersion objective. For each experiment, 200–400 non-transfected or transfected cells from random fields were quantified, and condensed or fragmented nuclei scored as apoptotic cells [38]. All analyses were performed by an experimenter blind to conditions.

### Lysotracker staining

HeLa cells transfected with either GFP or GFP-TFEB were plated into 35 mm imaging dishes (IBIDI, 81156) and treated with or without 50 μM FAC for 48 h. Cells were loaded with lysotracker Red DND-99 according to the manufacturer’s instructions (Invitrogen, L7528) in pre-warmed probe-containing medium for 10 min at 37 °C. Cells were washed twice with PBS before immediately imaging on a Leica TCS-SP5 confocal microscope using a 63 × 1.4 NA oil UV objective (HCX PLAPO CS) in a medium containing no phenol red. Cell shape was determined by either GFP or GFP-TFEB fluorescence, and the total number of lysosomes per cell was determined by ImageJ. The ROI enlarge function in ImageJ was set to −5 (ShrinkSelection macro in ij.jar) to determine the number of perinuclear lysosomes as previously described [40]. The number of peripheral lysosomes was calculated as total lysosomes minus perinuclear lysosomes. A total of 20 cells per condition and experiment were analyzed in this manner.

### Endolysosomal iron staining

For endolysosomal iron staining, we employed a modified sulfide-silver method using the FD RapidTimmStain Kit (FD Neurotechnologies, PK701) [11, 38, 41]. HEK293T cells were transfected on coverslips with either GFP or GFP-TFEB, and either left untreated or treated with 50 μM FAC for 48 h. Under these conditions, endocytosed iron is expected to be localized exclusively to endolysosomes, and iron staining is typically observed in the vicinity of the nucleus. Cells were washed in phosphate buffer and fixed with 2% glutaraldehyde in 0.1 M Na-cacodylate buffer (Sigma Aldrich, 70114) containing 0.1 M sucrose (pH 7.2) for 2 h at room temperature. Fixed cells were then rinsed with warm distilled water, followed by sulphidation with sulfide solution for 10 min, and three incubations in 0.1 M phosphate buffer for 5 min each. Development was performed using FD RapidTimmStain Kit components for 20 min at 30 °C in the dark. The reaction was stopped by transferring coverslips to warm distilled water, and cells protected from light were rinsed in distilled water three times for 5 min each. Coverslips were dehydrated in graduated ethanol solutions (50%, 75%, absolute ethanol) and mounted using Canada Balsam (Sigma Aldrich, C1795). Phase contrast images were acquired using an Olympus IX81 microscope (Olympus Corporation, Tokyo, Japan) using a ×100 objective. Cell shape was determined from phase contrast images, and the total number of iron-positive structures per cell quantified using NIH ImageJ software. The number of iron-positive peripheral lysosomes was calculated as total minus perinuclear iron-positive lysosomes. A total of 25 cells per condition and experiment were analyzed in this manner.

### Iron dequenching imaging and quantification

HEK293T cells were co-transfected with TRPML1-flag and mRFP, with TRPML1-T232P-flag and mRFP, or with TRPML2-RFP and pCMV, respectively. Iron dequenching assays were performed as previously described [11, 38]. Briefly, cells were incubated in Hanks balanced salt solution (10 mM HEPES, 140 mM NaCl, 5 mM KCl, 1.3 mM MgCl_2_, 2 mM CaCl_2_, 1 g/l D-glucose, pH 7.4) and incubated with 20 μM of the diacetate of Phen Green SK (Invitrogen, P14312), an iron-sensitive fluorescent indicator, for 20 min at 37 °C. Phen Green SK fluorescence after loading is dependent on the intracellular concentrations of both the indicator itself and of chelatable iron which quenches the fluorescence of the indicator. Therefore, after recording baseline fluorescence for 5–10 min, a non-fluorescent membrane-permeable iron chelator (2,2´-bipyridyl; 5 mM) was added in excess to remove the iron from the Phen Green SK indicator, which leads to an increase in fluorescence. An analog of 2,2´-bipyridyl (4,4´-bipyridyl, 5 mM) without iron chelating capacity was used as negative control.

Fluorescence measurements were performed on an Olympus IX81 microscope with a ×40 air objective. Green fluorescence of the Phen Green SK compound was excited at 488 nm, and emission collected through a 505 nm long-pass filter. Acquisition was performed once per minute so as to minimize photobleaching of the dye. For each experiment, an average of 30–40 cells were analyzed, with single-cell fluorescence determined from confined regions of interest. Since many variables including dye loading can contribute to variation of basal fluorescence of Phen Green SK, the normalized change of fluorescence (peak deltaF/F0) was employed as readout to estimate the change in cytosolic iron levels [11, 38].

### Acid phosphatase assay

HEK293T transfected with either GFP or GFP-TFEB were grown to sub-confluency in complete medium in 6-well plates. When cells were attached, FAC (50 μM) was added where indicated in medium containing 1% BSA. After 36 h, cells were washed three times in Hanks balanced salt solution and then incubated in 1 ml of medium containing 1% BSA for 12 h at 37 °C in the presence or absence of FAC (50 μM), NAADP-AM (100 nM) or Ned19 (1 μM) as indicated. Secreted and intracellular acid phosphatase activities were measured using the Acid Phosphatase Assay Kit (Sigma Aldrich, CS0740) according to manufacturer’s instructions and as previously described [17], and protein concentrations were determined using the BCA assay (Pierce Biotechnology).

### Statistical analysis

For each set of experiments, the sample size was chosen to ensure adequate power to detect variations. One-way ANOVA with Tukey’s *post hoc* test was employed, and the significance set at *p* < 0.05. Significance values for all data are indicated in the figure legends. Statistical analysis and graphs were performed using Prism software version 7.0 (GraphPad, San Diego, CA).

## Results

### Alterations in brain iron and copper content in G2019S-LRRK2 PD patients as compared to healthy controls

**Fig. 1:**
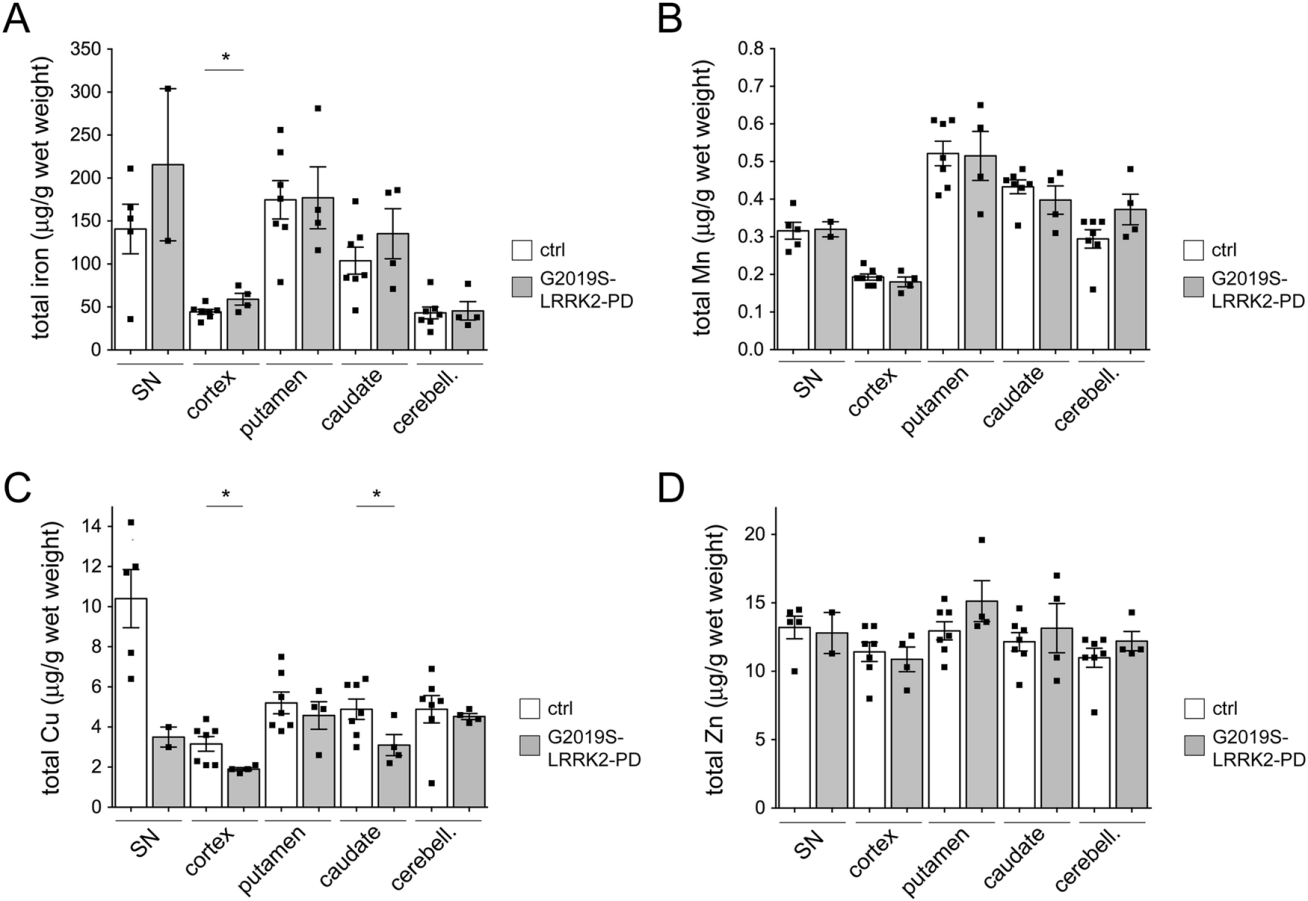
Region-specific alterations in total iron and copper content in the brains of G2019S-LRRK2 PD patients as compared to age-matched healthy controls. **A** Total iron content per wet weight of substantia nigra (SN), cortex, putamen, caudate, and cerebellum from seven age-matched healthy controls and four G2019S-LRRK2 PD patients. **B** As in **A**, but depicting total Mn content. **C** As in **A**, but depicting total Cu content. **D** As in **A**, but depicting total Zn content. Three independent tissue blocks from each brain region were analyzed, and analysis of each sample was performed in triplicates to obtain one mean value for each patient and brain region. Bars represent mean ± s.e.m. (*p < 0.05).

Sporadic PD patients display increased iron concentrations, especially in the nigrostriatal system, and excessive iron deposition is thought to contribute to oxidative stress and neuronal cell death [42]. Aberrant iron concentrations have also been observed in other brain regions such as the cortex where they may correlate with cognitive impairment [43, 44]. However, brain iron deposition in familial forms of PD such as those due to autosomal-dominant point mutations in LRRK2 has only been analyzed by in vivo imaging studies, with contradictory results obtained [45, 46]. To determine whether iron also accumulates in PD patients due to the G2019S-LRRK2 mutation as compared to healthy age-matched controls (Table 1), we measured total iron and other trace metal content in distinct brain areas by inductively coupled plasma mass spectrometry (ICP-MS). As previously described [47, 48], total iron levels in healthy controls were higher in the substantia nigra, putamen, and caudate as compared to cortex or cerebellum (Fig. 1A). Values of trace metal content per wet weight of tissue from the various brain areas were similar to those previously described, with no gender-dependent differences observed (not shown) [47, 48]. As compared to healthy controls, a significant increase in total iron was detected in the cortex of G2019S-LRRK2 PD patients, with a trend observed for substantia nigra and caudate (Fig. 1A). Determination of manganese or zinc levels from the same samples did not reveal differences between G2019S-LRRK2 PD patients and controls in any of the brain areas examined (Fig. 1B, D). However, a significant decrease in total copper content was observed in the caudate and cortex of G2019S-LRRK2 PD patients as compared to controls, with a trend observed for substantia nigra (Fig. 1C). These data indicate that identical to sporadic PD patients [49–52], G2019S-LRRK2 PD patients also display regional increases in iron and decreases in copper levels as compared to healthy age-matched controls.

**Table 1:** Demographic data for G2019S-LRRK2 Parkinson’s disease (G2019S-PD) and control patients.

	Sex	Age at death	Braak stage	PMI (h)
Control	F	56	0	14
Control	F	90	0	12.6
Control	M	78	0	6
Control	M	64	0	10
Control	M	86	0	7.25
Control	M	57	0	4.45
Control	F	51	0	2.75
G2019S-PD	F	77	3	**8**
G2019S-PD	F	92	4	7
G2019S-PD	F	69	5	12.3
G2019S-PD	M	85	3	17

Age, gender, disease severity according to the Braak staging system (Lewy body (LB) pathology in distinct brain areas) and postmortem interval (PMI) are indicated, with no significant differences between age at death and PMI between control and G2019S-LRRK2 PD patients (mean ± s.d.: age control 69 ± 15 versus G2019S-PD 80 ± 10; postmortem control 8 ± 4.3 versus G2019S-PD 11 ± 4.5). Please note that specific brain regions were not available from some patients.

Age-dependent increases in brain iron have been reported in humans as well as in animal models [53, 54]. We therefore next analyzed iron content in the cortex and cerebellum from young (3 months), middle-aged (9–11 months), and old (18–24) wild-type and G2019S-LRRK2 knockin mice [30]. A tendential age-dependent increase in iron content was observed in both wild-type and G2019S-LRRK2 knockin mice. However, and in contrast to human brain, no consistent region-specific increases in iron content were detected in G2019S-LRRK2 knockin mice as compared to controls (Fig. S1), at least as determined here by ICP-MS.

### Iron-mediated cytotoxicity is regulated by TRPML1 and TRPML2 channels

Since the G2019S-LRRK2 mutation did not cause detectable increases in total iron content, we next turned to cultured cells and chemically manipulated alterations in iron homeostasis in vitro. Treatment of cells with FAC causes endocytic Fe^3+^ uptake, which is followed by increased endolysosomal iron content, increased cytosolic oxidative stress, and cell death [38]. Endolysosomal TRPML1 channels can conduct iron [11] and may be activated by NAADP [55–57], a potent second messenger shown to function in many cell types [58, 59]. Therefore, we wondered whether modulation of TRPML1 channel activity may regulate iron-associated cytotoxicity.

**Fig. 2:**
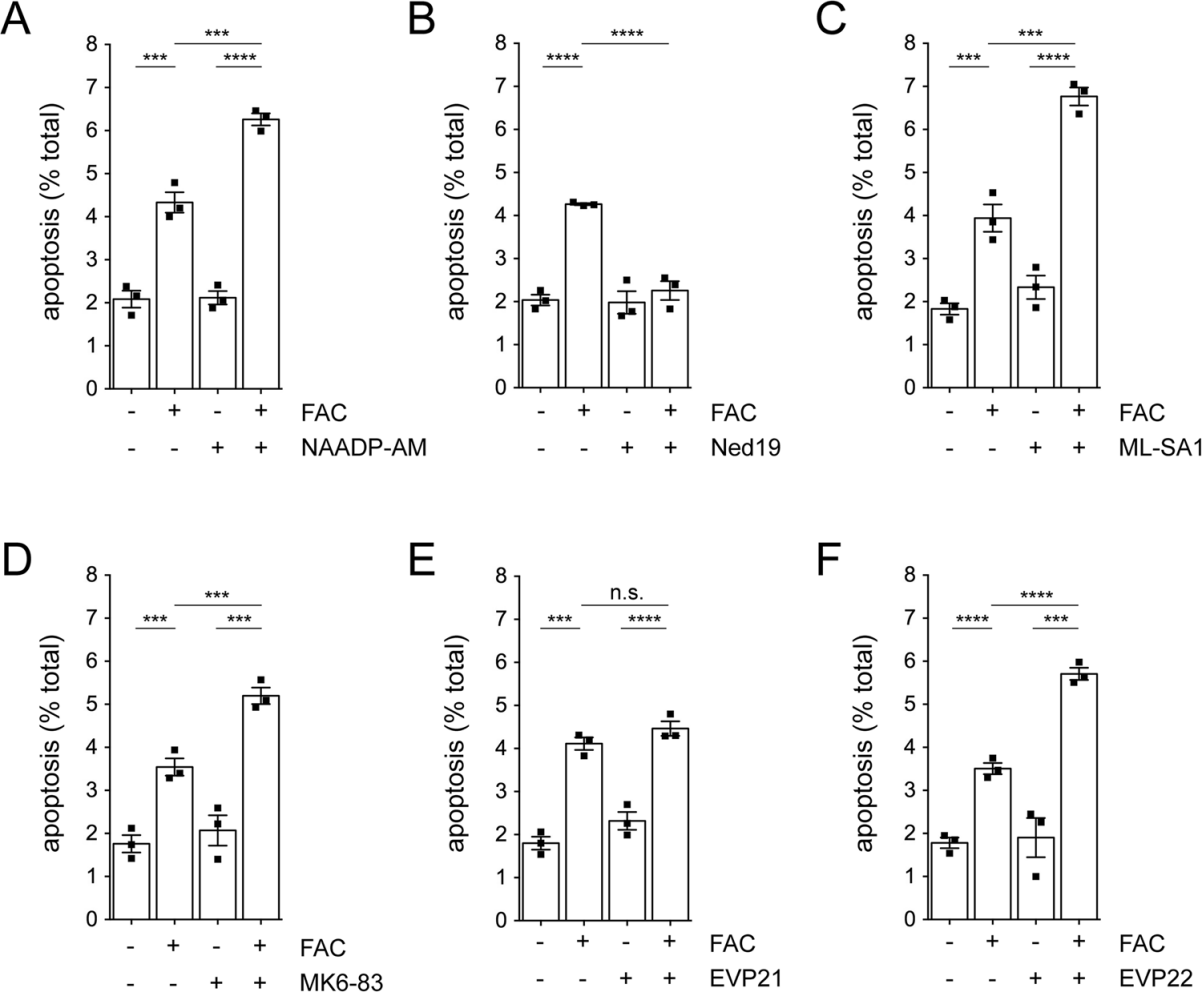
FAC-mediated apoptosis is potentiated by TRPML1 and TRPML2 activators. **A** HEK293T cells were treated with or without FAC (50 μM, 48 h) and with or without 100 nM NAADP-AM for the last 12 h as indicated. **B** As in **A**, but with or without Ned19 (1 μM, 12 h). **C** As in **A**, but with or without ML-SA1 (5 μM, 12 h). **D** As in **A**, but with or without MK6-83 (5 μM, 12 h). **E** As in **A**, but with or without EVP21 (5 μM, 12 h). **F** As in **A**, but with or without EVP22 (5 μM, 12 h). Bars represent mean ± s.e.m. (****p < 0.001; ***p < 0.005; n.s., non-significant).

Treatment of cells with FAC caused an increase in apoptosis which was potentiated by a membrane-permeable analog of NAADP (NAADP-AM) and reversed by Ned19, a highly selective NAADP antagonist [24] (Fig. 2A, B). Since NAADP also activates endolysosomal TPC channels [60], we employed a set of distinct agonists with exquisite selectivity towards the different TRPML channels [26–29]. The FAC-mediated increase in apoptosis was potentiated by ML-SA1 which targets all three TRPMLs (Fig. 2C). Potentiation was also observed with MK6-83 (TRPML1/3 agonist) and EVP22 (TRPML2 agonist), but not with EVP21 (TRPML3 agonist) (Fig. 2D–F). Expression of TRPML1 and to a lesser degree TRPML2 enhanced cell death due to iron overload, which was further potentiated by NAADP and blunted by Ned19, respectively (Fig. S2A, B). In addition, the different agonists potentiated the FAC-mediated cytotoxicity in TRPML-expressing cells according to their reported selectivities towards the various TRPML channels (Fig. S2C–F). These data indicate that TRPML1 and TRPML2 but not TRPML3 contribute to iron overload-mediated cytotoxicity.

**Fig. 3:**
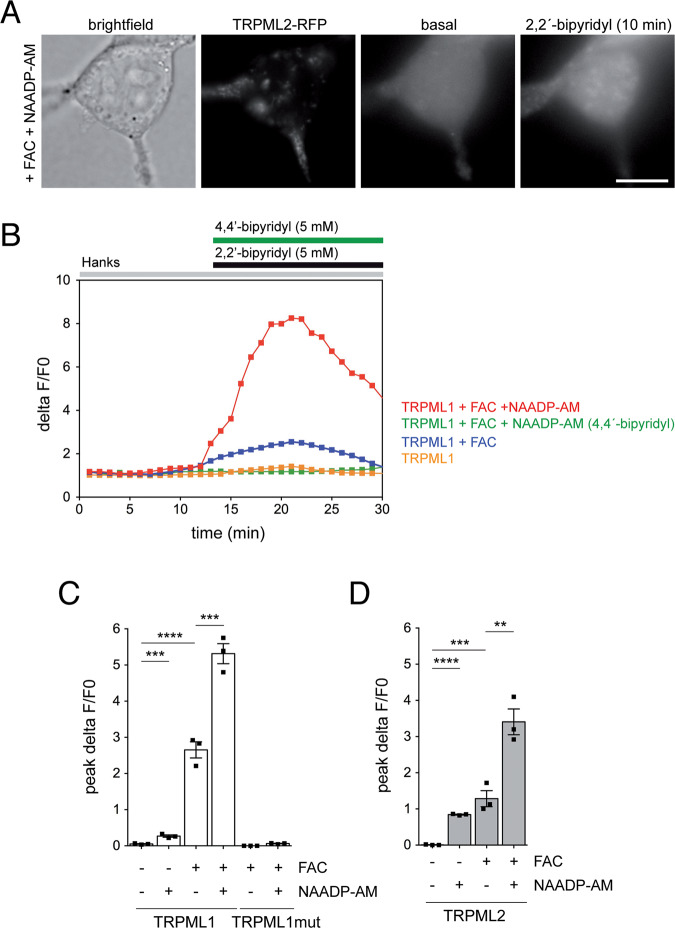
TRPML1 and TRPML2 channel-expressing cells display a FAC-induced increase in free cytosolic Fe^2+^ levels. **A** Representative example of dequenching of TRPML2-mRFP-expressing HEK293T cell upon incubation with FAC (50 μM, 24 h) and 100 nM NAADP-AM for the last 12 h. Cells were pre-loaded with the iron-sensitive dye Phen Green SK, followed by addition of the membrane-permeable transition metal chelator 2,2´-bipyridyl which chelates free cellular iron and causes a concomitant increase in Phen Green SK fluorescence. Scale bar, 10 μm. **B** Example of 2,2´-bipyridyl-induced normalized changes in fluorescence (deltaF/F0) in TRPML1-expressing cells (average of 30–40 cells per condition) in the absence or presence of FAC and NAADP-AM as indicated. Green trace shows the absence of fluorescence dequenching upon addition of 4,4´-bipyridyl, an analog of 2,2-bipyridyl which cannot bind iron. **C** Quantification of the 2,2´-bipyridyl-induced normalized change of peak fluorescence (peak deltaF/F0) in either wild-type or point-mutant TRPML1-expressing cells in the absence or presence of FAC and NAADP-AM as indicated. **D** Same as **C**, but in TRPML2-expressing cells. Bars represent mean ± s.e.m. (****p < 0.001; ***p < 0.005; **p < 0.01).

To determine whether the activation of TRPML1 or TRPML2 causes an increase in free cytosolic Fe^2+^ levels, we employed a fluorescence-based Fe^2+^ dequenching imaging method [11]. In cells expressing TRPML1, FAC treatment caused an increase in cytosolic Fe^2+^ levels in a manner potentiated by NAADP, which was not observed in cells expressing a point-mutant version of TRPML1 (TRPML1-T232P) unable to conduct iron [11], and a smaller FAC-induced increase in cytosolic Fe^2+^ was also observed in cells expressing TRPML2 (Fig. 3A–D). Thus, endolysosomal Fe^2+^ release via TRPML1 or TRPML2 channels increases cytosolic free Fe^2+^ levels to cause cytotoxicity under iron overload conditions.

### TFEB expression rescues iron-mediated cytotoxicity and causes exocytosis of iron-laden lysosomes

**Fig. 4:**
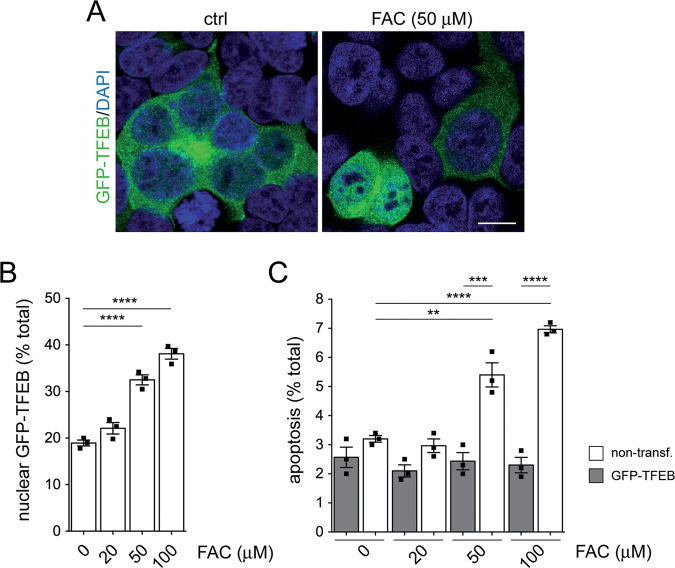
TFEB expression reverts apoptosis due to iron overload. **A** Representative example of HEK293T cells transfected with GFP-TFEB and with or without FAC treatment (50 μM, 48 h). Scale bar, 10 μm. **B** Cells were treated with increasing concentrations of FAC for 48 h as indicated. The percentage of cells with nuclear GFP-TFEB accumulation was quantified from 200–400 transfected cells per condition and experiment. **C** As in **B**, but quantification of apoptosis from 200–400 non-transfected versus transfected cells in the presence of increasing concentrations of FAC for 48 h. Bars represent mean ± s.e.m. (****p < 0.001; ***p < 0.005; **p < 0.01).

The TFEB regulates lysosomal biogenesis and lysosomal exocytosis by increasing the population of lysosomes ready to fuse with the plasma membrane and inducing the release of endolysosomal Ca^2+^ which can be mediated by TRPML1 [15–18]. Since TFEB overexpression is able to restore lysosomal morphology and function in various cellular and animal models of lysosomal storage diseases [15, 17, 61, 62], we wondered whether its expression may also rescue the endolysosomal iron overload-associated cytotoxicity. In TFEB-expressing cells, FAC treatment caused a dose-dependent translocation of GFP-TFEB to the nucleus (Fig. 4A, B) which was detectable as early as 12 h upon treatment (Fig. S3A, B) and was associated with the reversal of iron-mediated cell death (Fig. 4C, Fig. S3C).

**Fig. 5:**
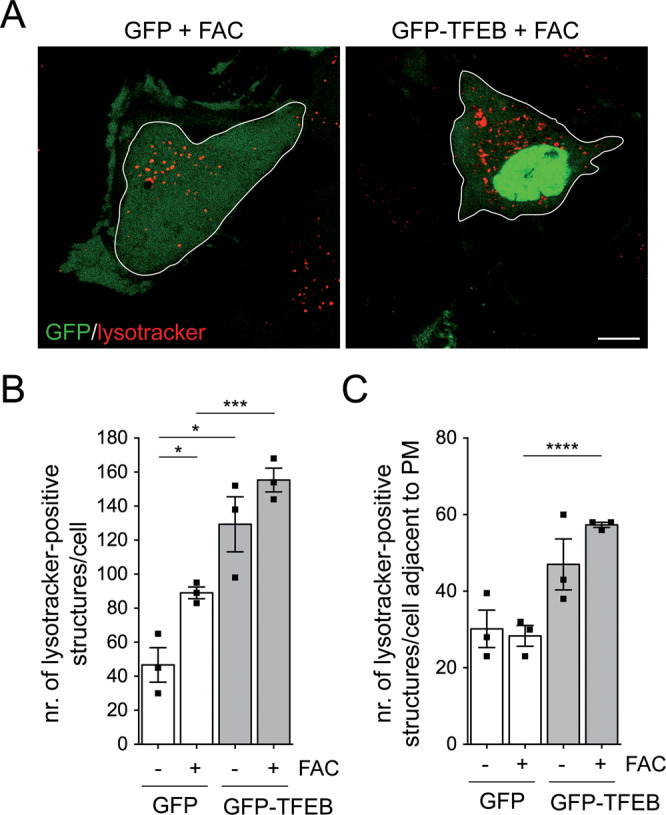
TFEB expression increases total and peripheral lysosomes in the absence and presence of iron overload conditions. **A** Example of HeLa cells transfected with either GFP or GFP-TFEB and treated with 50 μM FAC for 48 h before staining with lysotracker. Cell shape (white lines) was determined by GFP fluorescence. Scale bar, 10 μm. **B** Quantification of total lysotracker-positive structures per cell in cells expressing either GFP or GFP-TFEB and with or without FAC treatment as indicated. A total of 20 transfected cells per condition and experiment were quantified. **C** Same as in **B**, but quantification of peripheral lysotracker-positive structures per cell. Bars represent mean ± s.e.m. (****p < 0.001; ***p < 0.005; *p < 0.05).

**Fig. 6:**
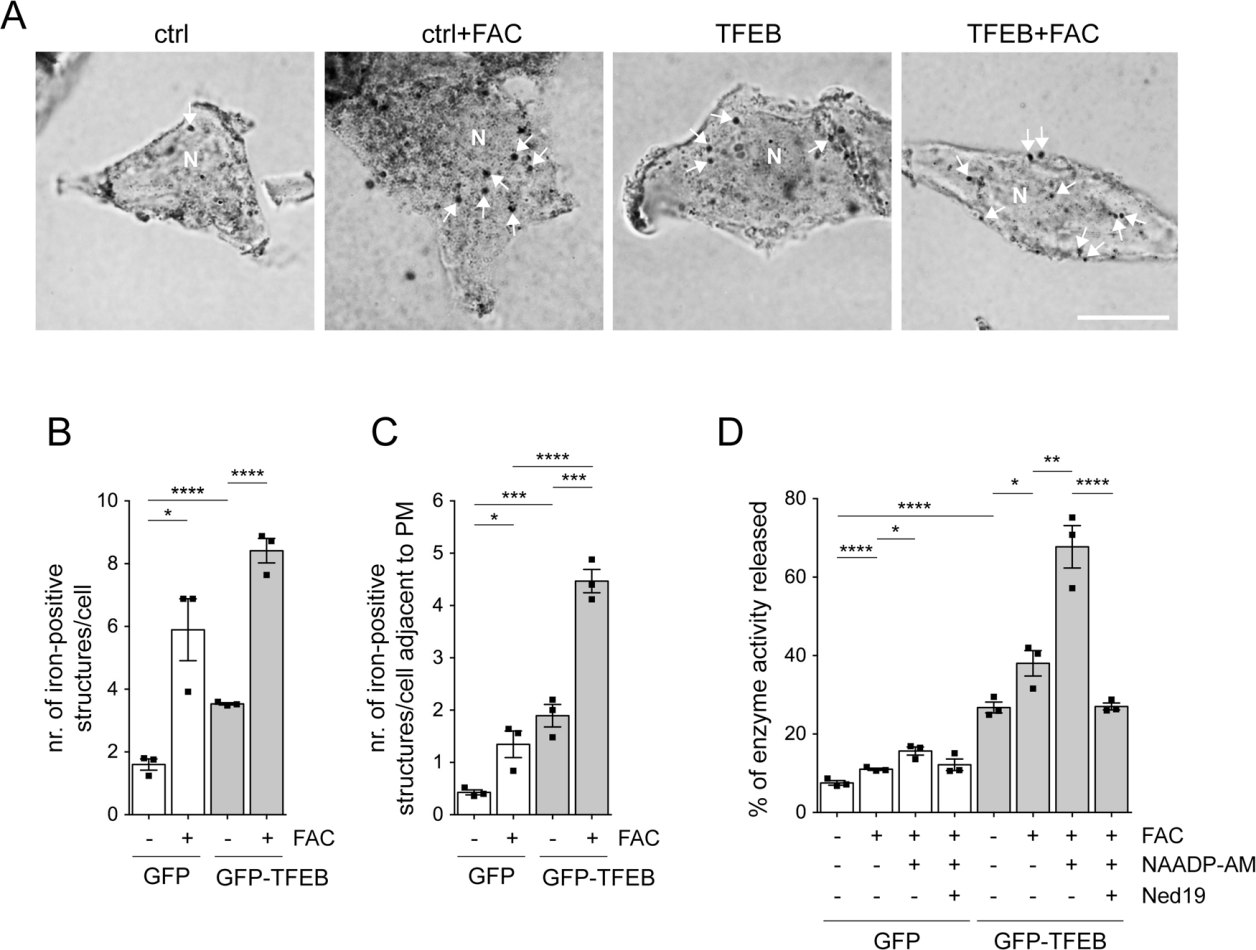
TFEB expression increases peripheral iron-positive endolysosomes and triggers lysosomal exocytosis. **A** Example of control GFP-expressing (ctrl) or GFP-TFEB-expressing HEK293T cells either without or with FAC treatment (50 μM, 48 h) as indicated, and stained for intracellular iron using the sulfide-silver method. Scale bar, 10 μm. **B** Quantification of the total number of iron-positive structures per cell in the absence or presence of FAC treatment as indicated. **C** Same as in **B**, but quantification of peripheral iron-positive structures per cell. **D** Activity of lysosomal enzyme acid phosphatase (percentage released as compared to total) was determined in culture medium from cells transfected with either GFP or GFP-TFEB, and treated with or without FAC (50 μM, 48 h), NAADP-AM (100 nM, 12 h) or Ned19 (1 μM, 12 h) as indicated. Bars represent mean ± s.e.m. (****p < 0.001; ***p < 0.005; **p < 0.01; *p < 0.05).

As assessed by lysotracker staining, FAC treatment in control GFP-expressing cells increased the total number of lysosomes without altering the pool of peripheral lysosomes (Fig. 5A–C). In contrast, TFEB expression increased both total and peripheral lysosomes (Fig. 5A–C). This was observed in the absence as well as the presence of FAC, indicating that TFEB expression was triggering lysosomal exocytosis also under iron overload conditions (Fig. 5B, C). To directly determine the number and localization of iron-containing lysosomes, we stained cells with the sulfide-silver method [11, 38, 41]. FAC-treated control cells displayed a significant increase in iron-positive lysosomes which were mostly localized to a perinuclear area (Fig. 6A–C). TFEB expression increased both total and peripheral iron-positive lysosomes in the presence of FAC (Fig. 6A–C). Therefore, and as determined by lysotracker staining in live cells and sulfide-silver staining in fixed cells, TFEB expression increases the pool of iron-laden peripheral lysosomes, indicative of lysosomal exocytosis.

A consequence of lysosomal exocytosis is the release of lysosomal enzymes into the cell culture medium[17]. Indeed, higher levels of secreted acid phosphatase were detected in cells expressing TFEB as compared to control cells (Fig. 6D). FAC treatment further increased the amount of lysosomal enzyme in the medium which was potentiated by NAADP and reverted by Ned19 (Fig. 6D). These data suggest that TFEB is able to trigger lysosomal exocytosis dependent on endolysosomal channels also under iron overload conditions.

### TFEB expression rescues iron-mediated cytotoxicity via calcium-dependent lysosomal exocytosis

**Fig. 7:**
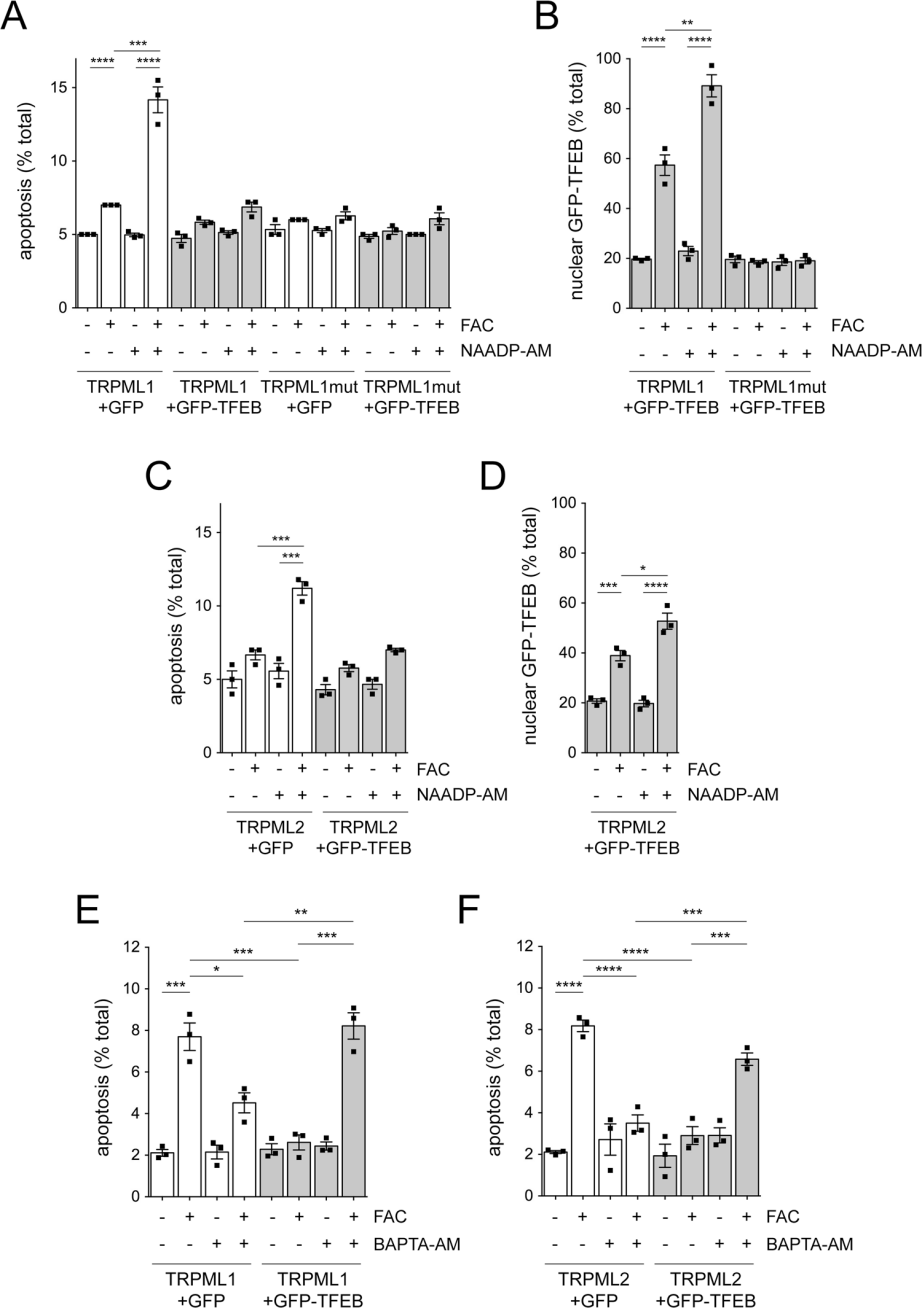
Rescue of iron overload-associated cytotoxicity by TFEB depends on TRPML1- and TRPML2-mediated calcium release. **A** HEK293T cells were co-transfected with the indicated constructs and treated with FAC (50 μM, 24 h) and with NAADP-AM (100 nM) for the last 12 h where indicated, and apoptosis quantified from co-transfected cells. **B** Same as in **A**, but quantification of nuclear translocation of GFP-TFEB from transfected cells. **C** Cells were co-transfected with the indicated constructs, and treated with FAC (50 μM, 24 h) and NAADP-AM (100 nM, 12 h) as indicated, and apoptosis quantified from co-transfected cells. **D** Same as in **C**, but quantification of nuclear translocation of GFP-TFEB from co-transfected cells. **E** Cells were co-transfected with TRPML1 and either GFP or GFP-TFEB and treated with FAC (50 μM, 24 h) and BAPTA-AM (5 μM, 2 h) where indicated, and apoptosis quantified from co-transfected cells. **F** Cells were co-transfected with TRPML2 and either GFP or GFP-TFEB and treated with FAC and BAPTA-AM as indicated, and apoptosis was quantified from co-transfected cells. Bars represent mean ± s.e.m. (****p < 0.001; ***p < 0.005; **p < 0.01; *p < 0.05).

Since lysosomal exocytosis mediated by TFEB depends on endolysosomal Ca^2+^ release [16–18], we asked whether the TFEB-mediated rescue of cell death due to iron overload was also dependent on increased intracellular Ca^2+^ levels. TRPML1 expression increased FAC-mediated cell death, and this was further potentiated by NAADP but not observed when expressing inactive point-mutant TRPML1 (TRPML1-T232P) (Fig. 7A). The FAC-induced cell death in TRPML1-expressing cells was completely prevented when co-expressing TFEB and correlated with TFEB nuclear translocation (Fig. 7A, B), and similar results were obtained in TRPML2-expressing cells (Fig. 7C, D). Importantly, the FAC-triggered cell death in TRPML1-expressing cells was blunted in the presence of BAPTA-AM, which is able to chelate calcium but also iron [63] (Fig. 7E, F). In contrast, the TFEB-mediated reversal of cell death due to iron overload in TRPML1-expressing cells was abolished in the presence of BAPTA-AM, and similar effects were observed in TRPML2-expressing cells (Fig. 7E, F). Therefore, the TFEB-induced reversal of cytotoxicity associated with iron overload depends on raised intracellular calcium levels via either TRPML1 or TRPML2, which is followed by lysosomal exocytosis.

## Discussion

The purpose of the present study was to probe for alterations in brain iron content in humans and in a mouse model of the most common form of familial PD, and to determine how iron overload-associated cytotoxicity may be regulated in vitro. ICP-MS was chosen as the method of choice for brain metal content determination, as it is associated with high sensitivity, exceptional linearity over up to nine orders of magnitude, and the ability to analyze multiple elements simultaneously [48]. Concentrations of iron, copper, manganese, and zinc in different brain regions from healthy human controls were in excellent agreement with those previously reported using the same analysis method [47]. The variability in metal content amongst age-matched individuals was also similar to previous findings [47]. We observed region-specific increases in iron content in G2019S-LRRK2 PD patients as compared to healthy age-matched controls, with a trend observed for substantia nigra and caudate, and a significant increase observed for cortex. No differences in manganese or zinc content were found in any of the brain regions analyzed. However, a significant decrease in copper content was detected in the caudate and cortex from G2019S-LRRK2 PD patients as compared to controls, with a trend observed for substantia nigra. Consistent region-specific increases in iron and decreases in copper levels have been previously reported for sporadic PD patients, and were not influenced by other variables such as sex or postmortem interval [51]. The link between increased iron and decreased copper levels in brain regions susceptible to neurodegeneration remains unclear but may involve the redistribution of these two metal ions, since higher iron and lower copper levels have been detected in individual neurons in susceptible brain regions from sporadic PD patients as compared to healthy controls [49]. Copper is a critical co-factor in a range of proteins including the protective cellular antioxidant superoxide dismutase 1 (SOD1) [64]. Copper depletion in various model systems reduces SOD1 activity and increases free radical production which can be normalized by copper supplementation [65]. In addition, studies in animal models of PD indicate that overexpression of SOD1 increases neuronal survival [66, 67]. Therefore, decreased copper levels may lead to reduced antioxidant capacity in regions susceptible to neurodegeneration [50], and future studies are warranted to investigate the crosstalk between iron and copper homeostasis in PD. Interestingly, a decrease in TRPML1-mediated endolysosomal calcium release has been reported in primary neurons from R1441C-LRRK2 but not from G2019S-LRRK2 knockin mice [68]. In addition, the zinc/copper ionophore clioquinol was able to rescue the lysosomal deficits mediated by R1441C-LRRK2 by mobilizing zinc into the lysosome [68]. It will be important to determine whether clioquinol is also able to restore lysosomal copper levels [69], and how this in turn may affect lysosomal iron levels. In addition, further studies are warranted to determine whether the presence of R1441C-LRRK2 or G2019S-LRRK2 may worsen the FAC-mediated cytotoxicity. In either case, our data show that region-specific increases in iron and decreases in copper content are not only associated with sporadic PD, but are also observed in PD patients due to the G2019S-LRRK2 mutation.

In contrast to human brain, no region-specific differences in iron or copper content were observed in G2019S-LRRK2 knockin mice as compared to controls, and iron content did not substantially increase with age in either G2019S-LRRK2 knockin or control mice. This is in contrast to a previous study reporting an increase in non-heme iron in the cortex of old as compared to young control mice [54]. Since there is no possibility to detect heme versus non-heme fractions of iron after mineralization when performing ICP-MS, we cannot exclude the possibility that non-heme iron content increases in the rodent brain in an age-dependent manner. In addition, and given the reported differences in age-dependent brain iron content amongst different mouse strains [53, 54], the lack of observed differences may be due to the C57BL/6 genetic background of the control and G2019S-LRRK2 knockin mice. Alternatively, differences between human and mouse as observed here may be due to the absence of neuromelanin in murine models. Neuromelanin is the main iron storage molecule in catecholaminergic neurons and may represent a toxic source of iron if the iron-binding capacity of this pigment becomes saturated [4, 70]. Therefore, it will be interesting to determine whether age-dependent alterations in human brain iron and copper content are recapitulated in a neuromelanin-producing rodent model, where TFEB overexpression has been reported to revert the resulting dopaminergic neurodegeneration [71]. Finally, and since proinflammatory stimuli trigger iron deposition in microglia from G2019S-LRRK2 knockin as compared to control mice [72], it is also possible that persistent neuroinflammation [73] contributes to the differences in iron and copper content in human G2019S-LRRK2 PD brains as determined here.

Lysosomes are critical for cellular iron homeostasis and are the first organelle to receive extracellular iron imported via the endocytic pathway. Once in its reduced form, Fe^2+^ can be released from the lysosome into the cytoplasm where it can enter the labile iron pool and give rise to oxidative stress followed by cell death [74]. We show here that TRPML1 and to a lesser degree TRPML2 function as endolysosomal Fe^2+^ release channels. Their activation by distinct compounds induces cytotoxicity under iron overload conditions, demonstrating an important role for these two channels as regulators of iron-mediated cell death. We further find that TFEB overexpression prevents such iron overload-associated cell death. The protective effect of TFEB expression correlates with an increase in lysosome biogenesis and lysosomal exocytosis which is dependent on intracellular Ca^2+^. Collectively, our data suggest that TRPML1/2 channels can conduct both Fe^2+^ and Ca^2+^. Under iron overload conditions, triggering TRPML1/2-mediated endolysosomal Fe^2+^ release is cytotoxic, whilst TRPML1/2-mediated Ca^2+^ release is required for the cytoprotective effects of TFEB. Therefore, either TFEB expression, or TRPML1/2 activation in conjunction with iron chelation, may be promising therapeutic approaches toward treating neurodegenerative diseases associated with iron dyshomeostasis.

## Supplementary information

Supplementary Material

reproducibility checklist

## Data Availability

Additional data can be found in the Supplementary Information. The remaining datasets are available from the corresponding author upon reasonable request.
