# Supplementary material for: Iron-induced cytotoxicity mediated by endolysosomal TRPML1 channels is reverted by TFEB
Source: Cell Death Dis. 2022 Dec 16;13(12):1047. doi: 10.1038/s41419-022-05504-2 (PMC9755144; doi:10.1038/s41419-022-05504-2)
Supplement: Supplementary file 1 — Supplementary Material [file 41419_2022_5504_MOESM1_ESM.docx]

**
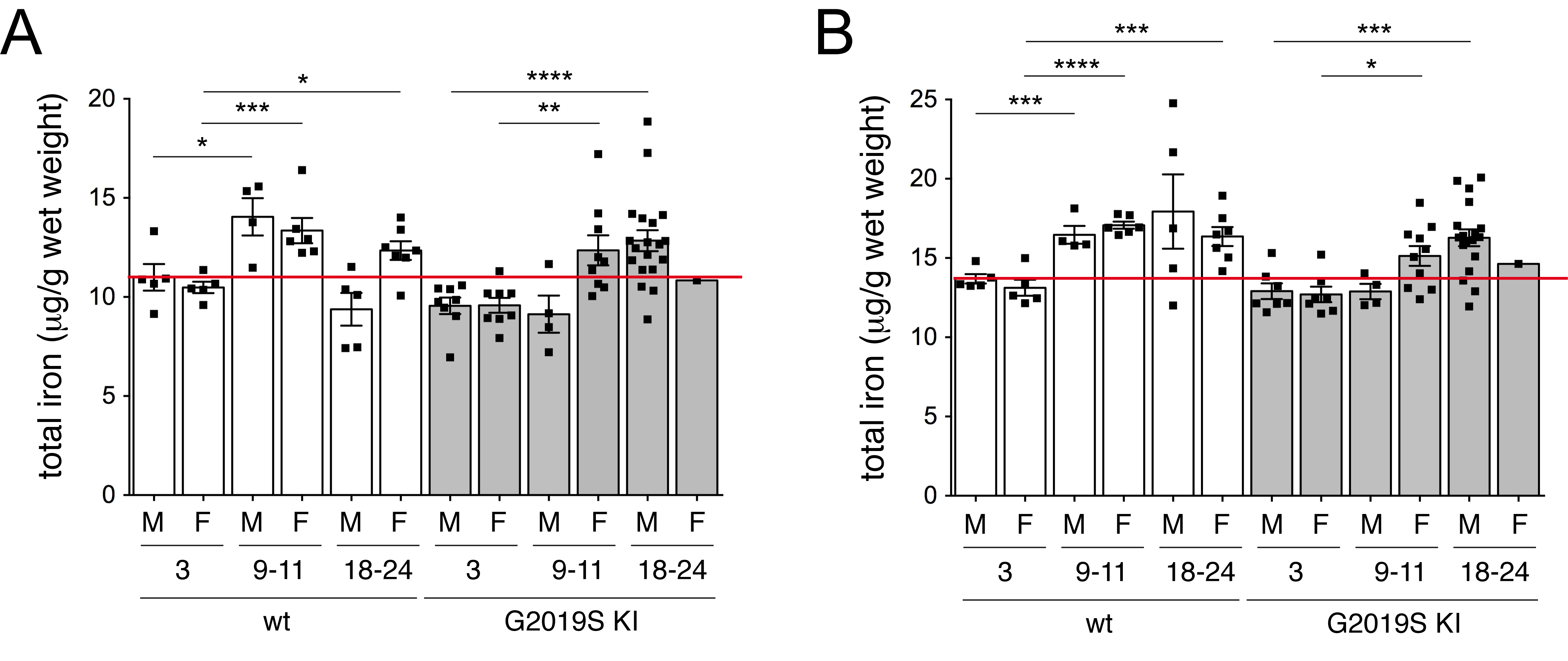
**

**Figure S1.** Iron content in the brains of G2019S-LRRK2 knockin mice as compared to age-matched wildtype controls. (**A**) Total iron content per wet weight of cortex from young (3 months), middle-aged (9-11 months) and old (18-24 months) wildtype (wt) and G2019S-LRRK2 knockin (G2019S KI) mice. (**B**) As in (A), but total iron content per wet weight determined from cerebellum. Analysis of each sample was performed in triplicates to obtain one mean value for each mouse and brain region. Bars represent mean ± s.e.m. (****p < 0.001; ***p < 0.005, **p < 0.01; *p < 0.05).


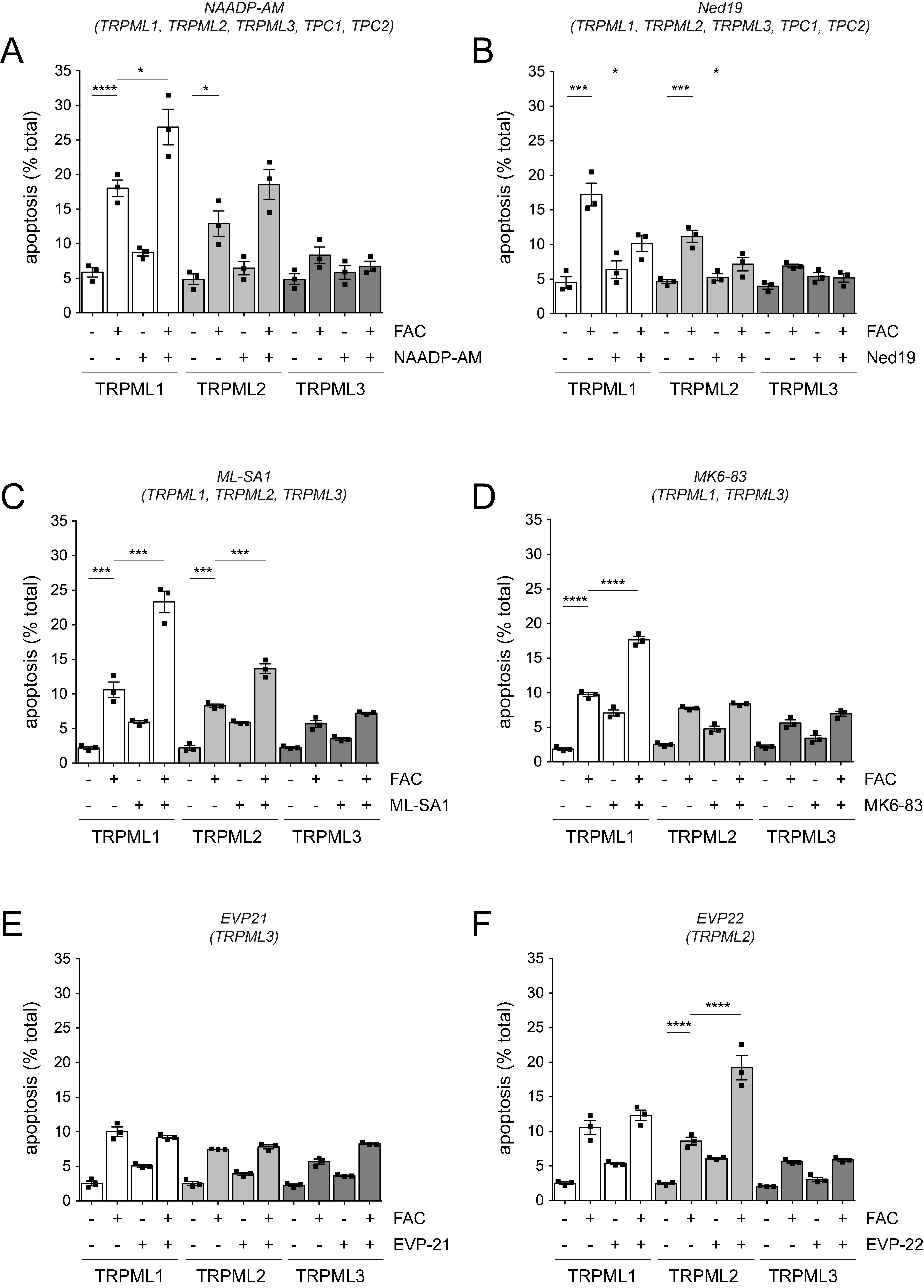


**Figure S2.** TRPML activators potentiate FAC-mediated apoptosis according to their selectivies for the different TRPML channels. (**A**) HEK293T cells were transfected with either TRPML1, TRPML2 or TRPML3, and treated with or without FAC (50 μM, 24 h) and with or without NAADP-AM (100 nM) for the last 12 h as indicated. (**B**) As in (A), but cells treated with Ned19 (1 μM, 12 h). (**C**) As in (A), but cells treated with ML-SA1 (5 μM, 12 h). (**D**) As in (A), but cells treated with MK6-83 (5 μM, 12 h). (**E**) As in (A), but cells treated with EVP-21 (5 μM, 12 h). (**F**) As in (A), but cells treated with EVP-22 (5 μM, 12 h). Bars represent mean ± s.e.m. (****p < 0.001; ***p < 0.005; *p < 0.05).


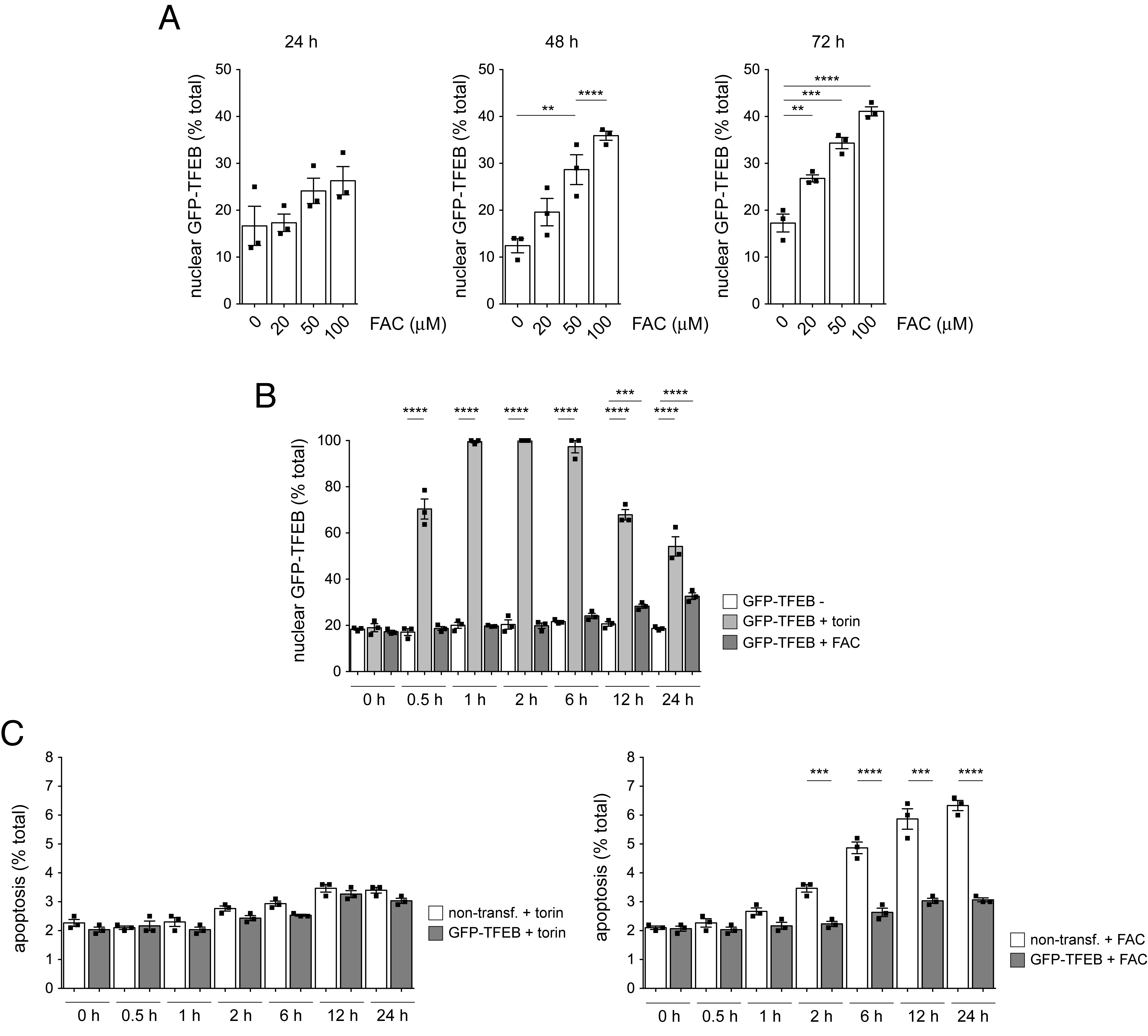


**Figure S3.** FAC causes a dose- and time-dependent nuclear translocation of GFP-TFEB. (**A**) HEK293T cells were transfected with GFP-TFEB and treated with increasing concentrations of FAC for either 24 h, 48 h or 72 h. Nuclear GFP-TFEB accumulation was quantified from 200-400 transfected cells per condition and experiment. (**B**) HEK293T cells were transfected with GFP-TFEB and treated with 50 μM FAC or 100 nM torin as a positive control for TFEB nuclear translocation for the indicated amounts of time, and nuclear translocation quantified from transfected cells. (**C**) As in (B), but quantification of apoptosis from 200-400 non-transfected versus transfected cells in the presence of 50 μM FAC for the indicated amounts of time. Bars represent mean ± s.e.m. (****p < 0.001; ***p < 0.005; **p < 0.01).
